# Spin-optoelectronic devices based on hybrid organic-inorganic trihalide perovskites

**DOI:** 10.1038/s41467-018-07952-x

**Published:** 2019-01-10

**Authors:** Jingying Wang, Chuang Zhang, Haoliang Liu, Ryan McLaughlin, Yaxin Zhai, Shai R. Vardeny, Xiaojie Liu, Stephen McGill, Dmitry Semenov, Hangwen Guo, Ryuichi Tsuchikawa, Vikram V. Deshpande, Dali Sun, Z. Valy Vardeny

**Affiliations:** 1 Department of Physics & Astronomy, University of Utah, Salt Lake City, UT 84112 USA; 2 College of Optical Sciences, University of Arizona, Tucson, AZ 85721 USA; 3 National High Magnetic Field Laboratory, Tallahassee, FL 32310 USA; 4 Department of Physics & Astronomy, Louisiana State University, Baton Rouge, LA 70803 USA; 5 Department of Physics, North Carolina State University, Raleigh, NC 27695 USA

## Abstract

Recently the hybrid organic-inorganic trihalide perovskites have shown remarkable performance as active layers in photovoltaic and other optoelectronic devices. However, their spin characteristic properties have not been fully studied, although due to the relatively large spin-orbit coupling these materials may show great promise for spintronic applications. Here we demonstrate spin-polarized carrier injection into methylammonium lead bromide films from metallic ferromagnetic electrodes in two spintronic-based devices: a ‘spin light emitting diode’ that results in circularly polarized electroluminescence emission; and a ‘vertical spin valve’ that shows giant magnetoresistance. In addition, we also apply a magnetic field perpendicular to the injected spins orientation for measuring the ‘Hanle effect’, from which we obtain a relatively long spin lifetime for the electrically injected carriers. Our measurements initiate the field of hybrid perovskites spin-related optoelectronic applications.

## Abstract (web summary)

Hybrid organic-inorganic trihalide perovskites can make remarkable optoelectronic devices but their spin characteristics are less investigated. Here Wang et al. show spin-polarized carriers injection into methylammonium lead bromide films with long lifetime and realize spin LEDs and spin valves.

## Introduction

Finding materials for spintronics applications that simultaneously possess strong spin–orbit coupling (SOC) for efficient spin manipulation, long-spin relaxation time for efficient spin transport, and strong photoluminescence (PL) emission for spin-optoelectronic applications, has been a challenge. Hybrid organic–inorganic trihalide perovskites (OITP) are emerging semiconductors which have recently attracted intense research interest for optoelectronic applications^1,2^. The OITP such as MAPbX_3_, where MA is methylammonium and X is a halogen, may combine the advantages of both organic and inorganic semiconductors for spin-optoelectronic applications. These compounds are grown using solution-based low-temperature methods, which lead to easy fabrication and flexible device engineering^3^. Moreover, they have synthetically tunable electronic and optical properties with strong PL emission. Also due to the heavy atoms in their building blocks, the OITP possess a relatively large SOC. Indeed, the recently obtained Rashba-splitting in the OITP^4,5^, the optical spin selection rules^6,7^, and magnetic field effect^8^ caused by strong SOC have initiated spintronics research avenue for these compounds. So far, however no spin-related optoelectronics device application based on OITP has been reported. Thus, demonstration and studies of spintronic devices based on this class of semiconductors are intriguing.

In order to realize spin-related optoelectronic devices based on OITP, electrical spin injection from ferromagnet (FM) electrodes into OITP films should be demonstrated. Here, we successfully demonstrate spin injection from FM electrode into MAPbBr_3_, detected by both optical and electrical means. For the optical detection, we have used the circularly polarized electroluminescence (EL) emission from MAPbBr_3_ film in a spin light emitting diode (spin-LED) device based on a FM electrode. Spin-LED devices, first realized using III–V semiconductors^9,10^, were considered to be strong evidence for confirming spin injection into various semiconductors^11–13^. The confirmed spin injection ability shows that the OITP successfully overcome the conductivity mismatch problem^14^. In addition, we also demonstrate electrical spin injection into OITP using spin valve (SV) devices based on MAPbBr_3_ interlayer, where giant magneto-resistance (GMR) of up to about 25% has been achieved. Importantly, we also observed the “electrical Hanle effect” in the SV devices, from which we obtained at cryogenic temperatures a spin lifetime, *τ*_s_ = 936 ± 23 ps for the injected spin 1/2 holes in MAPbBr_3_.

## Results

### Optical spin injection and Hanle effect in MAPbBr_3_

**Fig. 1:**
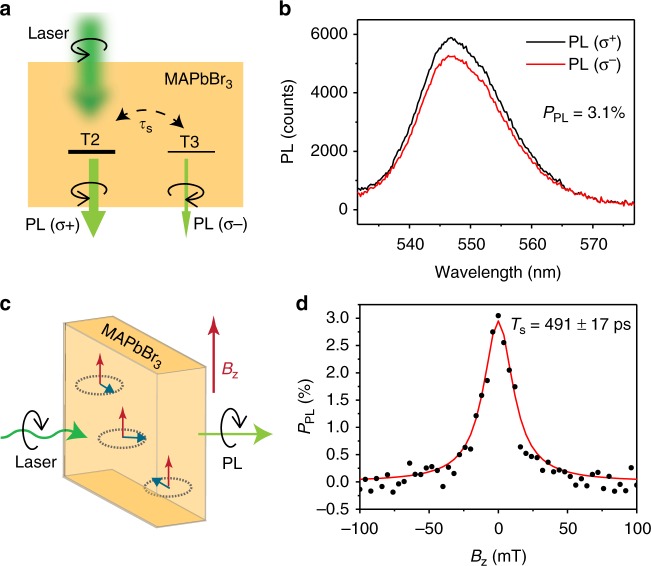
Circular polarization of the PL and optical Hanle effect in MAPbBr_3_. **a** Schematic of the circularly polarized excitation (*σ*^+^) and polarized PL emission components, PL(*σ*^+^) and P(*σ*^−^) in MAPbBr_3_. T2 and T3 are two exciton states that can emit circularly polarized PL and have spin-dependent optical transitions near the MAPbBr_3_ optical gap. Here PL(*σ*^+^) > PL(*σ*^−^), as marked by a heavier bar underneath the T2 label. Spin relaxation process couples the two exciton states with spin lifetime,*τ*_s_. **b** PL(*σ*^+^) and PL(*σ*^−^) emission spectra in MAPbBr_3_ at steady state measured at 10 K. The degree of circular polarization, *P*_PL_ = [PL(*σ*^+^) − PL(*σ*^−^)]/[PL(*σ*^+^) + PL(*σ*^−^)] of 3.1% is obtained. **c** Schematic set-up for measuring the optical Hanle effect. A magnetic field, *B*_z_ perpendicular to the PL light propagation direction is applied, which causes spin precession that leads to diminishing circular polarization of PL. **d** The *P*_PL_(*B*_z_) response measured at 10 K. The red solid line is a fit using Eq. (1), from which we obtained an effective exciton spin lifetime, *T*_s_ = 491 ± 17 ps

To demonstrate spin injection from FM electrodes, we first studied circularly polarized PL emission in MAPbBr_3_ thin film at low temperature, in order to show that it maintains “optical spin alignment”^15^. We prepared polycrystalline films of MAPbBr_3_ by spin casting (see Methods). Scanning electron microscopy (SEM) illustrated an average grain size of around 100 nm in the film, whereas the X-ray-diffraction (XRD) pattern was similar to that in the literature (Supplementary Fig. 1 & Supplementary Note 1)^16^. The films were excited using circularly polarized (*σ*^+^) cw laser beam at 532 nm, and we measured the circular PL components, PL(*σ*^+^) and PL(*σ*^−^) (Fig. 1a) to obtain the degree of circular polarization, which is defined as *P*_PL_ = [PL(*σ*^+^) − PL(*σ*^−^)]/[PL(*σ*^+^) + PL(*σ*^−^)] at 10 K; we obtained *P*_PL_ of 3.1% (see Fig. 1b). The exciton binding energy in the orthorhombic phase of MAPbBr_3_ is about 30 meV^17^ so that the majority of the photoexcitations at steady state conditions at 10 K are excitons. The photogenerated excitons may populate four closely spaced states. These are three triplets, T1–T3, where T2 and T3 emit circularly polarized PL, and a dark (D) singlet exciton^18,19^. The non-zero *P*_PL_ validates the optical spin selection rules in MAPbBr_3_, which allows optical orientation and detection of spin-polarized excitations, as in other direct gap semiconductors such as GaAs^20^.

The polarization degree, *P*_PL_ is determined by the two spin-polarized excitons (T2 and T3) with population *N*^+(−)^ at steady state, where $P_{{\mathrm{PL}}} = \frac{{N^ + - N^ - }}{{N^ + + N^ - }}$. *P*_PL_ is related to the exciton spin lifetime, *τ*_s_ and lifetime *τ* by the relation: $P_{{\mathrm{PL}}} = \frac{\eta }{{1 + \tau /\tau _{\mathrm{s}}}}$^15^, where *η* is the initial polarization ratio after excitation. Considering the long exciton lifetime (of the order of tens ns) in MAPbBr_3_^2,21^ (Supplementary Fig. 2 & Supplementary Note 2), we conclude from the obtained *P*_PL_ value that *τ*_s_ cannot be very short.

When an external magnetic field, *B*_z_ is applied in the direction perpendicular to the spin orientation, the exciton spin precesses around *B*_z_ direction, as shown in Fig. 1c; this leads to quenching of *P*_PL_ (called optical Hanle effect). Figure 1d illustrates that *P*_PL_ indeed decreases with *B*_z_, which is traditionally described by the relation^22^:

1 $$P_{{\mathrm{PL}}}\left( {B_{\mathrm{z}}} \right) = \frac{{P_{{\mathrm{PL}}}(B_{\mathrm{z}} = 0)}}{{1 + (\omega _{\mathrm{L}}T_{\mathrm{s}})^2}}$$

where *ω*_L_ = *μ*_B_*g*_ex_*B*_z_/ℏ is the Larmor frequency. We have measured the *g*-factor value, *g*_ex_ of exciton in MAPbBr_3_ by magnetic circular dichroism (Supplementary Fig. 3 & Supplementary Note 3) to be *g*_ex_ = 1.254. In Eq. (1), $T_{\mathrm{s}} = 1/\left( {\frac{1}{\tau } + \frac{1}{{\tau _{\mathrm{s}}}}} \right)$ is the exciton effective spin lifetime. The red solid line in Fig. 1d is a fit using Eq. (1), from which we obtained *T*_s_ = 491 ± 17 ps in MAPbBr_3_ at 10 K. Taking *τ* = 15 ns^4^, we calculate from the measured *T*_s_ the value *τ*_s_ = 508 ± 17 ps for the exciton spin lifetime in MAPbBr_3_.

### Spin-LED device based on MAPbBr_3_

**Fig. 2:**
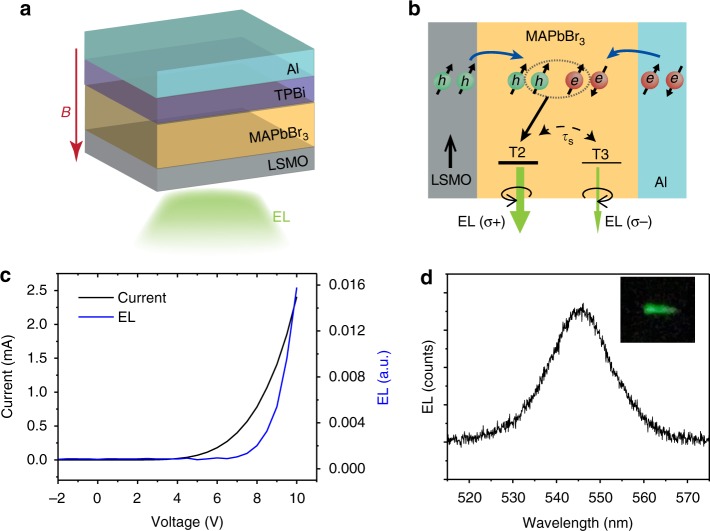
Spin-LED device based on MAPbBr_3_. **a** Schematic of the spin-LED device structure. The “half metal” LSMO serves as FM anode that is capable of injecting spin-polarized holes into the MAPbBr_3_ interlayer. The TPBi molecule thin film serves as the electron transport layer capped by an Al cathode. **b** Working principle of the spin-LED. Spin-polarized holes injected by the FM anode form e–h pairs with electrons injected by the nonmagnetic electrode according to the “optical spin selection rule”, which subsequently form spin-polarized excitons, T2 that emit circularly polarized electroluminescence, EL(*σ*^+^). Due to spin relaxation, T3 state may be also populated emitting EL(*σ*^−^). Here EL(*σ*^+^) > EL(*σ*^−^), as marked by a heavier bar underneath the T2 label. **c** Typical *I*–*V* and EL–*V* responses of the MAPbBr_3_-based spin-LED measured at 10 K. **d** The resulting EL spectrum; the picture in the inset shows the green EL emission from the device

Since MAPbBr_3_ shows circularly polarized PL emission, we may use it to probe spin injection from FM electrode by studying circularly polarized EL emission in a spin-LED device. We have therefore fabricated MAPbBr_3_ spin-LED based on the half metal FM electrode La_0.63_Sr_0.37_MnO_3_ (LSMO), which has nominally 100% spin polarization at the Fermi energy^23^. A thin MAPbBr_3_ film was spin coated on the LSMO substrate, which serves as an anode that injects spin-polarized holes. As a cathode we used an Al film coated on top of a thin layer of the organic small molecule, 2,2′,2″-(1,3,5-benzinetriyl)-tris (1-phenyl-1-H-benzimidazole) (TPBi), which serves as the electron transport layer. The device structure is illustrated in Fig. 2a. An external magnetic field was applied perpendicular to the electrodes, which is aligned along the propagation direction of the collected EL emission. Figure 2b shows the working principle of the spin-LED device; spin-polarized holes are injected from the ferromagnetic LSMO, while unpolarized electrons are injected from the non-magnetic Al electrode. Due to the relatively large exciton binding energy of 30 meV at 10 K, the injected e–h pairs form excitons according to the optical selection rules, then emit EL light (see Fig. 2b). Because of the imbalance in the spin populations of the injected holes, EL emission shows circular polarization.

Typical electrical characteristics response of the fabricated MAPbBr_3_ spin-LED device is shown in Fig. 2c. A clear rectifying behavior starting from a bias voltage, *V* = 5 Volt is seen. The EL–*V* response shows a turn-on voltage at 8 Volt; bright green light can be seen from an area of 0.5 mm × 3 mm (Fig. 2d inset). At *V* = 9 Volt and 10 K, we measured a strong EL emission band at 548 nm (Fig. 2d), which is consistent with the PL spectrum (Fig. 1b), and with traditional LEDs based on MAPbBr_3_^2^.

**Fig. 3:**
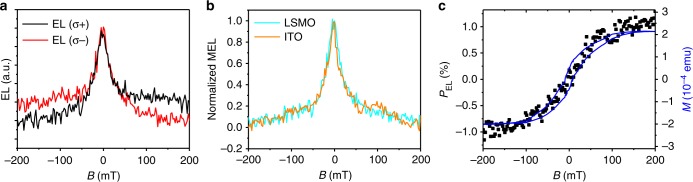
Circularly polarized electroluminescence emission from MAPbBr_3_ spin-LED. **a** EL(*σ*^+^) and EL(*σ*^−^) circularly polarized EL emission as a function of an applied magnetic field measured at 10 K and applied bias of 9 V. **b** The sum of the right and left circular polarized EL(*B*) response of the spin-LED, compared with MEL(*B*) response of a traditional LED with no FM electrodes, where the LSMO was replaced by indium tin oxide (ITO). **c** The degree of circular polarization in the EL(*B*) response. The blue solid line is the magnetization hysteresis loop of the LSMO electrode measured using SQUID magnetometry

The two circularly polarized EL components, EL(*σ*^+^) and EL(*σ*^−^) were measured when the out-of-plane magnetic field aligns the LSMO magnetization, as seen in Fig. 3a. A clear difference between EL(*σ*^+^) and EL(*σ*^−^) responses versus *B* is obtained. We note that the EL(*B*) response is a superposition of two components. (i) An intrinsic, symmetric magneto-EL (MEL) response, which is the same for the EL(*σ*^+^) and EL(*σ*^−^) responses (Fig. 3b). This response is due to a spin-mixing process within the e–h pairs^8^, and is the same as in an ordinary MAPbBr_3_-based LED (with no FM electrodes) that we also fabricated (see Fig. 3b). (ii) An asymmetric MEL(*B*) response which is different for the EL(*σ*^+^) and EL(*σ*^−^) emissions. We calculated the EL degree of circular polarization, *P*_EL_ using the relation *P*_EL_$= \frac{{{\mathrm{EL}}(\sigma ^ + ) - {\mathrm{EL}}(\sigma ^ - )}}{{{\mathrm{EL}}(\sigma ^ + ) + {\mathrm{EL}}(\sigma ^ - )}}$; its response versus magnetic field *B* is plotted in Fig. 3c. A maximum *P*_EL_ value of 0.8% is obtained at *B* larger than 150 mT. For comparison purpose, we also plot in Fig. 3c the out-of-plane magnetization *M*(*B*) response of LSMO electrode. It is clear that the obtained *P*_EL_(*B*) response follows the magnetization response of LSMO. This unambiguously proves that the circular polarization of the EL is caused by spin injection from the LSMO electrode, since *P*_EL_(*B*) is proportional to the spin polarization of the injected holes from LSMO electrode, which, in turn is related to LSMO magnetization response. A slight deviation, Δ*P*(*B*) of the *P*_EL_(*B*) response from the *M*(*B*) response of the LSMO electrode is seen at large *B*. This is caused by contribution from field induced Zeeman splitting^11^, and magnetic circular dichroism of the LSMO electrode. These responses were measured separately to be smaller than 0.1%, in agreement with the deviation response. (Supplementary Fig. 4 & Supplementary Note 4).

Similar to the circular polarization degree, *P*_PL_ in MAPbBr_3_ films discussed above, *P*_EL_ of the spin-LED can be expressed by the relation:

2 $$P_{{\mathrm{EL}}} = \frac{{N^ + - N^ - }}{{N^ + + N^ - }} = P_{\mathrm{s}}\frac{\eta }{{1 + \tau /\tau _{\mathrm{s}}}} = P_{\mathrm{s}}P_{{\mathrm{PL}}}$$

where *P*_s_ is the spin injection efficiency of the FM electrode, *η* is the initial spin polarization in MAPbBr_3_. Assuming *η* is the same for PL and EL measurements, from the obtained *P*_EL_ and *P*_PL_ expressions we get $\frac{{P_{{\mathrm{EL}}}}}{{P_{{\mathrm{PL}}}}} = P_{\mathrm{s}} = 26\%$ for the LSMO ferromagnetic electrode at 10 K. In fact, *η* for the EL emission should be smaller than for PL emission at resonance excitation, namely *η*_EL_ < *η*_PL_, since the spin aligned injected holes in the spin-LED may lose their spin alignment before forming e–h pairs. Therefore, 26% is a lower limit for the LSMO spin injection efficiency. Nevertheless, this large value is surprising when taking into account the existence of the seemingly unsurmised conductivity mismatch between a metallic FM and a semiconductor, which mostly prevents spin injection^14^. This is especially true for the perovskite-based spin-LED in which we found that the spin injection efficiency is one order of magnitude higher than the value in GaAs-based spin-LEDs^11,24^. We speculate that the efficient spin injection into the MAPbBr_3_ could be attributed to its large SOC, which is manifested by the obtained surface Rashba splitting^5^ that suppresses the conductivity mismatch, and, in turn improves the spin injection at the FM/MAPbBr_3_ interface^14^.

### Spin valve device based on MAPbBr_3_

**Fig. 4:**
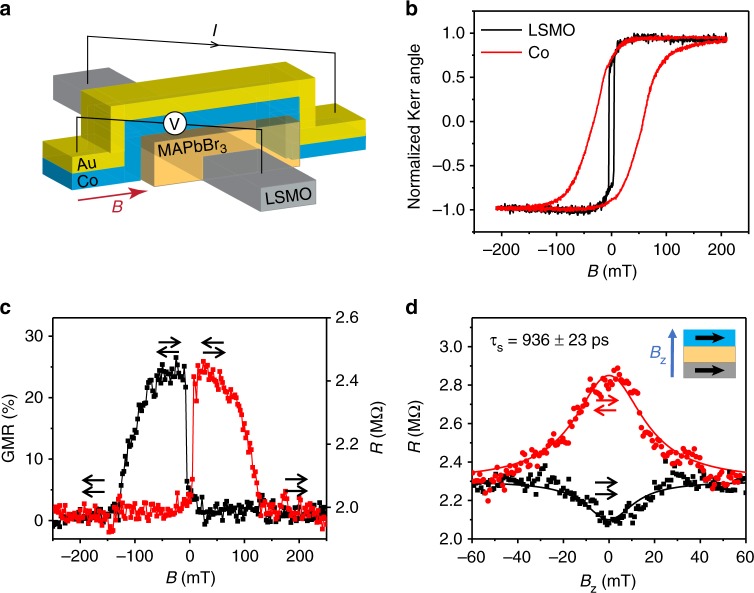
Giant magnetoresistance (GMR) and Hanle effect in MAPbBr_3_-based spin valve. **a** Schematic of a LSMO/MAPbBr_3_/Co spin valve device structure. **b** MOKE(*B*) response of the LSMO and Co ferromagnetic electrodes as measured in-situ in the SV device at 10 K. **c** GMR(*B*) response of the spin-valve measured at 10 K and applied bias voltage, *V* = 0.1 V. The obtained maximum GMR value (GMR_max_) is 25%. The red and black lines represent magnetic field sweep up and down, respectively. The arrows show the mutual magnetization direction of the two FM electrodes. **d** Hanle effect of the GMR measured when a magnetic field, *B*_z_ perpendicular to the FM electrodes is applied, at both parallel and antiparallel magnetization configurations. The solid lines are fits using Eq. (3), from which a spin lifetime *τ*_s_ = 936 ± 23 ps is extracted

We have also measured spin injection into MAPbBr_3_ film using electrical detection in a SV device. Figure 4a shows the SV device structure which is composed of two FM electrodes, namely a LSMO film grown on SrTiO_3_(001) substrate and an evaporated cobalt film, with MAPbBr_3_ film as spacer layer deposited using the same method as for the spin-LED device discussed above. An in-plane magnetic field *B* was applied to manipulate the magnetization orientation of the two FM electrodes. Magnetization hysteresis loops of both LSMO and Co electrodes were recorded in situ, using the magneto-optics Kerr effect (MOKE) measured by a Sagnac interferometer^25^. As shown in Fig. 4b, at 10 K we measured the coercive field, *B*_c1_ = 5 mT for LSMO and *B*_c2_ at around 75 mT for Co. Upon sweeping *B*, the relative magnetization orientation of the two FM electrodes changes from parallel (P) to antiparallel (AP) configuration, and vice-versa upon changing the field sweeping direction. This leads to change in the device resistance, *R*, with *R*_P_ < *R*_AP_; which is called giant magnetoresistance (GMR). The obtained magnetoresistance (MR) ratio is then calculated from the relation: MR = (*R*_AP_ − *R*_P_)/*R*_P_. The MR(*B*) response of a SV device based on MAPbBr_3_ measured at 10 K is shown in Fig. 4c. We obtained a substantial maximum GMR, GMR_max_ of 25%, indicating an effective spin injection into the MAPbBr_3_ interlayer film, which is consistent with our spin-LED response. Possible artefacts such as tunneling magnetoresistance (TMR), tunneling anisotropic magnetoresistance (TAMR), and anisotropic magnetoresistance (AMR) have been ruled out by control experiments. (Supplementary Figs. 6 & 7, Supplementary Note 6 & 7).

In order to cross-check our results, we conducted the electrical Hanle effect measurements on the same SV. A perpendicular magnetic field, *B*_z_ was applied to the SV device when the FM electrodes magnetization are in parallel or antiparallel configuration. Under this condition, the injected spin in the MAPbBr_3_ interlayer from one FM electrode precesses with Larmor frequency: *ω*_L_ = *gμ*_B_*B*_z_/ℏ while the carriers drift to the other FM electrode. This spin precession induces change in the relative angle between the spin polarization of injected carriers and the magnetization direction of the detecting FM electrode, thus causing change in device resistance. The precession angle, *θ*_p_ of the spin carriers that arrive at the opposing electrode is determined by the relation: *θ*_p_ = 2*πt*_trans_/*t*_p_, where *t*_p_ is spin precession period, and *t*_trans_ is the carriers transit time (or time of flight, TOF). In disordered materials such as the OITP films, carrier transport is dispersive as demonstrated by TOF measurements^26^, and this causes a broad distribution of *t*_trans_ value. Consequently, the spin polarization of injected spin aligned carriers averages out at the detecting electrode (spin dephasing), which quenches the measured GMR of the SV device^27,28^. Figure 4d shows that GMR_max_ value vanishes with increasing *B*_z_ for both parallel and antiparallel SV configuration. The observed Hanle effect unambiguously demonstrates spin injection and transport in the OITP interlayer; consequently our measurements show in fact GMR type response. As a control experiment, after the Hanle measurements were performed, the in-plane MR-loop was repeated to confirm that the magnetization state of the electrodes was not tilted or flipped by perpendicular field.

To extract additional information about the spin lifetime in the MAPbBr_3_ interlayer the electrical Hanle response, *R*(*B*_z_) may be fit using the 1-D spin drift-diffusion model^28^:

3 $$R(B_{\mathrm{z}}) \propto \mathop {\int }\limits_0^\infty \frac{1}{{\sqrt {4\pi Dt} }}{\mathrm{exp}}\left( { - \frac{{d^2}}{{4Dt}}} \right)\cos \left( {\omega _{\mathrm{L}}t} \right)\exp \left( { - \frac{t}{{\tau _{\mathrm{s}}}}} \right){\mathrm{d}}t$$

In Eq. (3), *D* is the spin diffusion coefficient, *d* is the MAPbBr_3_ film thickness (*d* = 207 nm here). *τ*_s_ and *ω*_L_ = *μ*_B_*gB*_z_/ℏ are the spin lifetime and Larmor frequency of the spin 1/2 injected carriers. Since holes are the injected carriers in our SV device type, we use in Eq. (3) the *g*-factor for holes: *g*_h_ = 0.33^6^. Using Eq. (3) to fit the Hanle response, we obtain *τ*_s_ = 936 ± 23ps and *D* = 0.21 ± 0.08 cm^2^ s^−1^ for the holes in MAPbBr_3_ at 10 K. This large spin lifetime contributes to the long spin diffusion length of around 220 nm in MAPbBr_3_ (Supplementary Fig. 8 & Supplementary Note 8). It is interesting that the obtained *τ*_s_ for holes is larger than that for excitons.

## Discussion

In this work, we presented the study of spintronic devices based on OITP polycrystalline films. Successful spin injection from FM metallic electrodes into OITP has been demonstrated. The spin lifetime for both excitons and holes in MAPbBr_3_ is surprisingly long despite the large SOC; this indicates that the OITP semiconductor family could be promising candidates for spintronics applications.

## Methods

### Sample preparation

The ferromagnetic LSMO thin films were grown on SrTiO_3_ (001) substrates by pulsed laser deposition (PLD) and patterned by wet-etch optical lithography. The LSMO electrodes were cleaned and re-used multiple times. The MAPbBr_3_ overlayer was deposited by the spin coating method inside a N_2_ filled glove box (O_2_/H_2_O < 1 ppm). The MAPbBr_3_ precursor solution was obtained by mixing PbBr_2_ and MABr (molar ratio = 1:1.1) in dimethyl sulfoxide (DMSO) at a concentration of 0.5 M. The perovskites solutions were stirred overnight at 50 °C before use. To prepare the perovskite thin film, the solutions were spin-coated on the O_2_ plasma treated substrates at 4000 rpm. To achieve pinhole-free microscopic structure for the MAPbBr_3_ film, chloroform solvent was drop-casted onto the perovskite film for a nanocrystal pinning process during spin coating (as described in ref. ^2^). Finally, the perovskite films were annealed on a hot plate at 100 °C for 30 min.

### Device fabrication

For the various devices, MAPbBr_3_ thin film was spin coated onto the bottom LSMO electrode. After cooling down to ambient temperature, the samples were transferred back to the vacuum chamber for e-beam evaporation of other layers. For the spin-LED device, the bottom LSMO electrode was patterned as 3 × 5 mm strips. Following the MAPbBr_3_ film deposition, a 10 nm TPBi film was deposited as the electron transport layer. Subsequently, a 100 nm aluminum film was coated as top electrode in a crossbar configuration. The typical device area was 0.5 × 3 mm. For the SV device, the bottom LSMO electrode was patterned as 0.2 × 5 mm strips. 15 nm cobalt film followed by 30 nm gold were capped as the second ferromagnetic electrode in a crossbar configuration, using a shadow mask. The typical device area in this case was 200 × 200 μm.

### Device characterization

The out-of-plane magnetization curve of LSMO electrode in spin-LED device is measured by SQUID magnetometer at 10 K. On the other hand, magnetic hysteresis loop of two ferromagnetic electrodes is measured by a home-built Sagnac interferometer with a static DC Kerr rotation sensitivity of 20 nrad, and spot size of about 1 μm. The MOKE responses of both LSMO and Co electrodes were measured at 10 K in a perovskite-based SV configuration. This unique technique has enabled us to measure the magnetization properties of the spin-polarized interface in situ rather than bulk Co and LSMO electrodes outside the SV device. The EL and PL were measured by a silicon detector. The transport measurements were performed in a closed-cycle refrigerator with temperature ranging from 10 to 300 K by a standard four points method with Keithley 236 power supply and Keithley 2000 multimeter with an in-plane magnetic field *B*.

## Supplementary information

Supplementary Information

Peer Review File

## Data Availability

The data that support the findings of this study are available from the corresponding author on reasonable request.
