# Supplementary material for: Spin-optoelectronic devices based on hybrid organic-inorganic trihalide perovskites
Source: Nat Commun. 2019 Jan 10;10:129. doi: 10.1038/s41467-018-07952-x (PMC6328620; doi:10.1038/s41467-018-07952-x)
Supplement: Supplementary file 1 — Supplementary Information [file 41467_2018_7952_MOESM1_ESM.docx]

**Supplementary Information**

**Spin-Optoelectronic Devices Based on Hybrid Organic-Inorganic Tirhalide Perovskites**

Jingying Wang^1^, Chuang Zhang^1^, Haoliang Liu^1^, Ryan McLaughlin^1^, Yaxin Zhai^1^, Shai R. Vardeny^2^, Xiaojie Liu^1^, Stephen McGill,^3^ Dmitry Semenov,^3^ Hangwen Guo^4^, Ryuichi Tsuchikawa^1^, Vikram V. Deshpande^1^, Dali Sun^1,5,*^, Z. Valy Vardeny^1,*^

^1^ *Department of Physics & Astronomy, University of Utah, Salt Lake City, UT, 84112, USA*

^2^ *College of Optical Sciences, University of Arizona, Tucson, AZ, 85721, USA*

^3^ *National High Magnetic Field Laboratory, Tallahassee, FL 32310, USA*

^4^ *Department of Physics & Astronomy, Louisiana State University, Baton Rouge, LA 70803, USA*

^5^ *Department of Physics, North Carolina State University, Raleigh, NC 27695, USA*

^*^To whom correspondence should be addressed: dsun4@ncsu.edu, val@physics.utah.edu.

**Supplementary Figures**


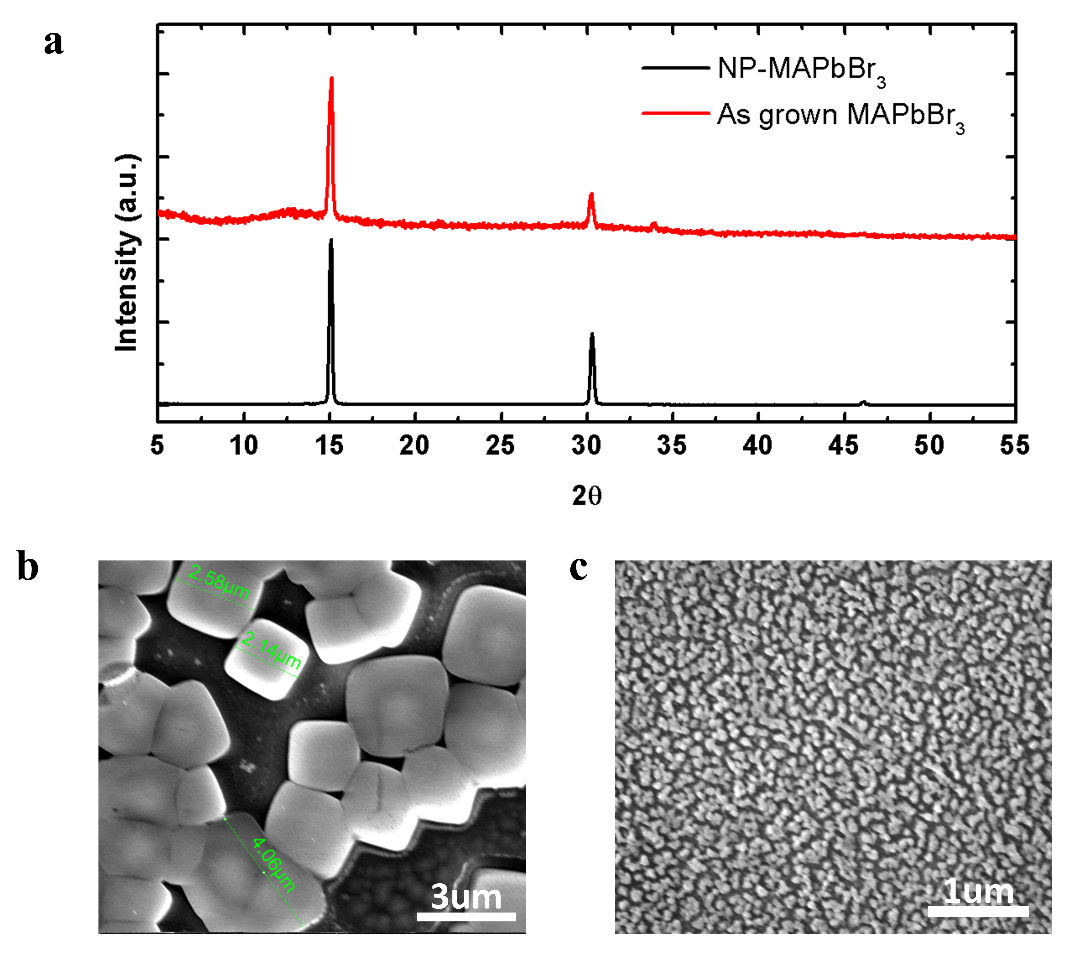


**Supplementary Figure 1 | Thin film Characterization a,** XRD spectra of as-grown MAPbBr_3_ and nanocrystal pining MAPbBr_3_ thin film. **b**, SEM image of as-grown MAPbBr_3_ film. **c**, SEM image of nanocrystal pining MAPbBr_3_ film.

**
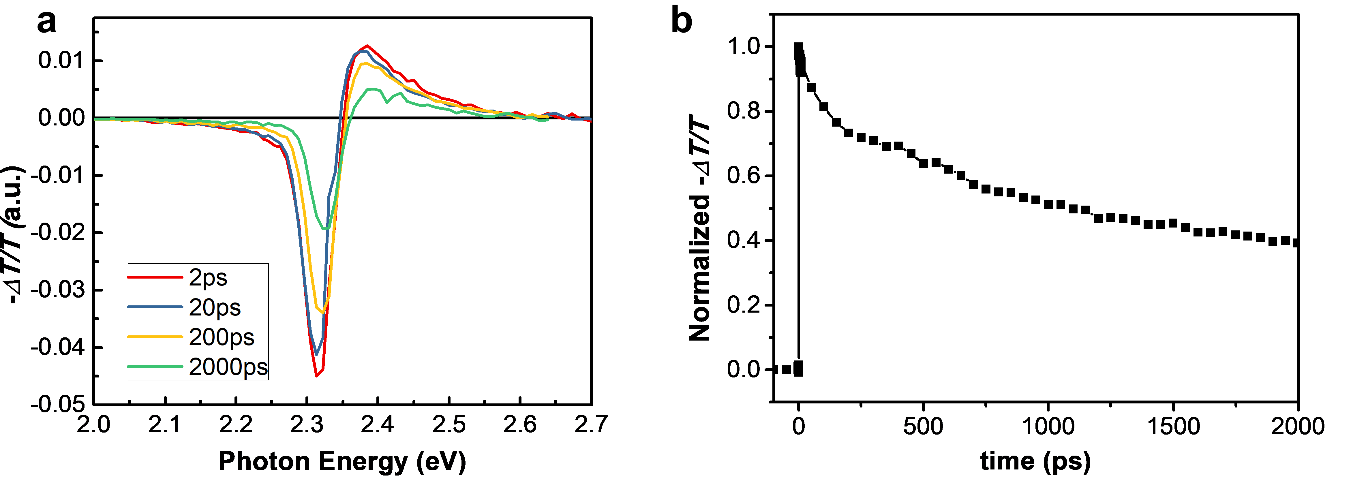
**

**Supplementary Figure 2 | Picosecond transient photoinduced absorption (t-PA) spectroscopy of the MAPbBr_3_ film at *T*=80K.** **a,** *ΔT(λ,t)* spectra at various time delays between the pump and probe pulses. The positive feature is due to photoinduced absorption (PA), whereas the negative feature is due to photo-bleaching (PB). **b,** The transient decay dynamics of the PB band at 2.31 eV.

**
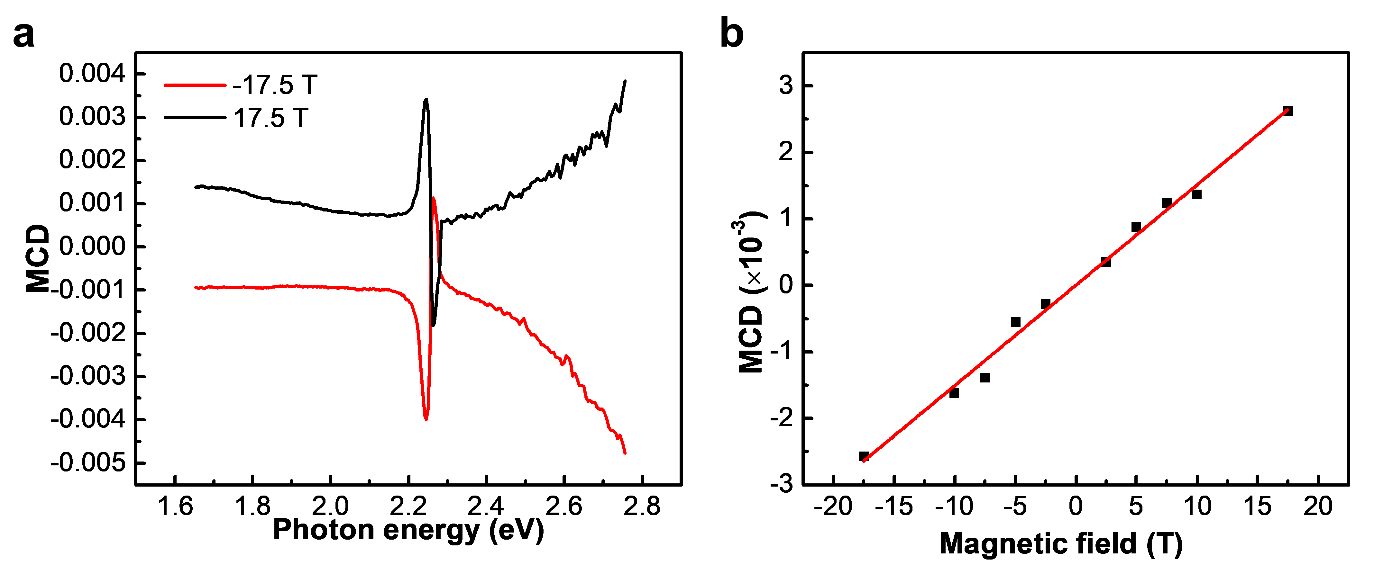
**

**Supplementary Figure 3 | Magnetic circular dichroism (MCD) of MAPbBr_3_.** **a,** MCD spectra of MAPbBr_3_ thin film measured at 5K with applied magnetic field of 17.5 T (black) and -17.5 T (red), respectively. **b,** The magnetic field dependence of MCD, from which the value of *g*-factor = 1.254 for the excitons in MAPbBr_3_ has been extracted.


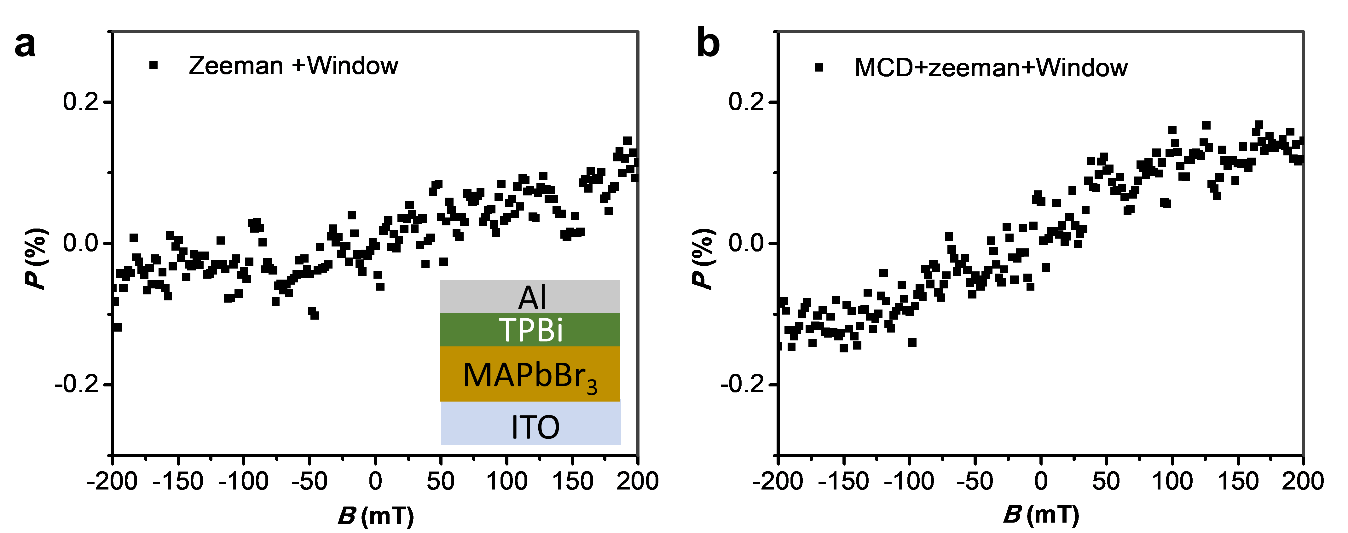


**Supplementary Figure 4 | Zeeman splitting and MCD response vs. magnetic field in a regular LED device based on MAPbBr_3_. a**, EL circular polarization measured in a traditional MAPbBr_3_-based LED device with nonmagnetic electrode, measured under the same conditions as for the spin-LED. The obtained circular polarization originates from field induced circular polarization in MAPbBr_3_ as well as MCD of the cryostat window. **b**, MCD response of the LSMO layer and cryostat window measured at 10K using the circular polarization of the PL emission in MAPbBr_3_ film.

**Supplementary Figure 5| Inversion symmetry breaking at MAPbBr_3_ surface.** Schematic illustrations of SHG, THG and three-photon induced PL (TP-PL) emissions generated in the bulk and surface, respectively of a MAPbBr_3_ crystal using multiple photon microscopy (MPM) technique. **(b)** and **(d)** MPM emission spectra inside the MAPbBr_3_ crystal and on its surface, respectively. The MPM is excited by a femtosecond (100 fs) laser at wavelength of 1550 nm. SHG signal (i.e., emission between 750 nm to 800 nm) is only observed on the surface of the MAPbBr_3_ crystal (panel d inset), and this shows lack of inversion symmetry at the surface.


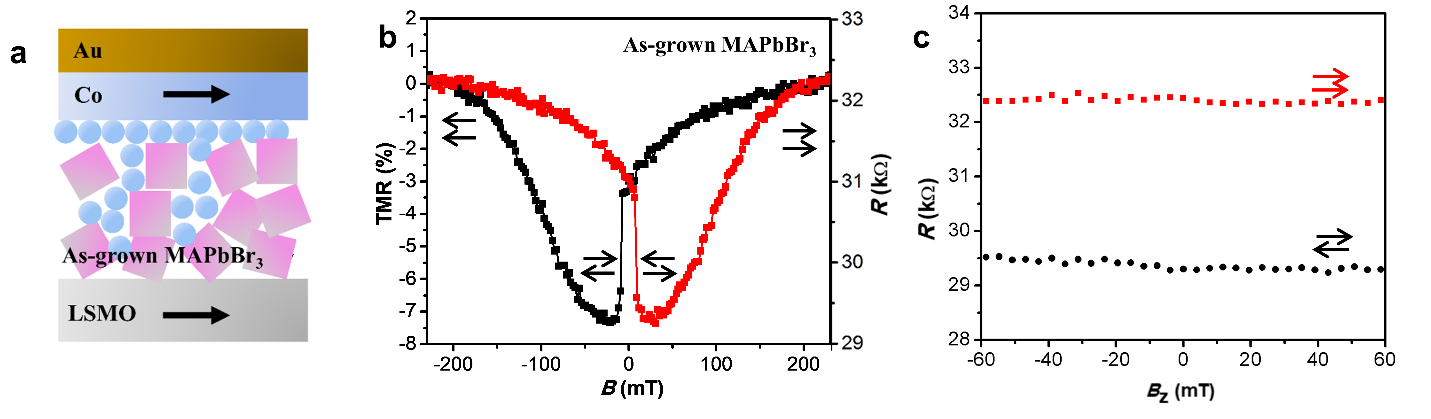


**Supplementary Figure 6 | Tunneling magnetoresistance in SV based on ‘as-grown’ MAPbBr_3_. a**, Schematic diagram of LSMO/MAPbBr_3_/Co SV, which is in fact a magnetic tunnel junction. Interdiffusion of Co atoms into the as-grown MAPbBr_3_ thin film leads to the occurrence of pinholes in the interlayer film. **b**, TMR(*B*) response in SV device based on as-grown MAPbBr_3_ interlayer, measured at 10 K and 0.1 V. The obtained maximum TMR value (TMR_max_) is 8%. **c**, No Hanle effect has been measured in the TMR-based device.


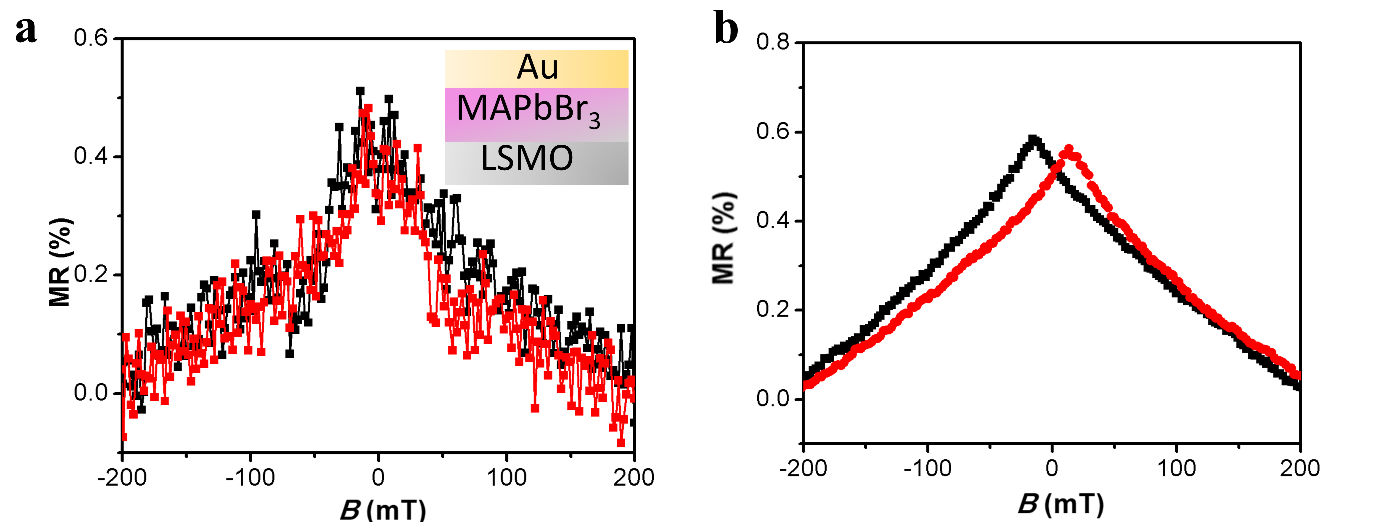


**Supplementary Figure 7 | Control experiments that rule out possible artefacts in the MAPbBr_3_-based spin valve devices. a**, MR(*B*) response measured in LSMO/MAPbBr_3_/Au structure. No TAMR was observed. **b**, AMR(*B*) response of 0.6% measured for LSMO bottom electrode at 10K.

**
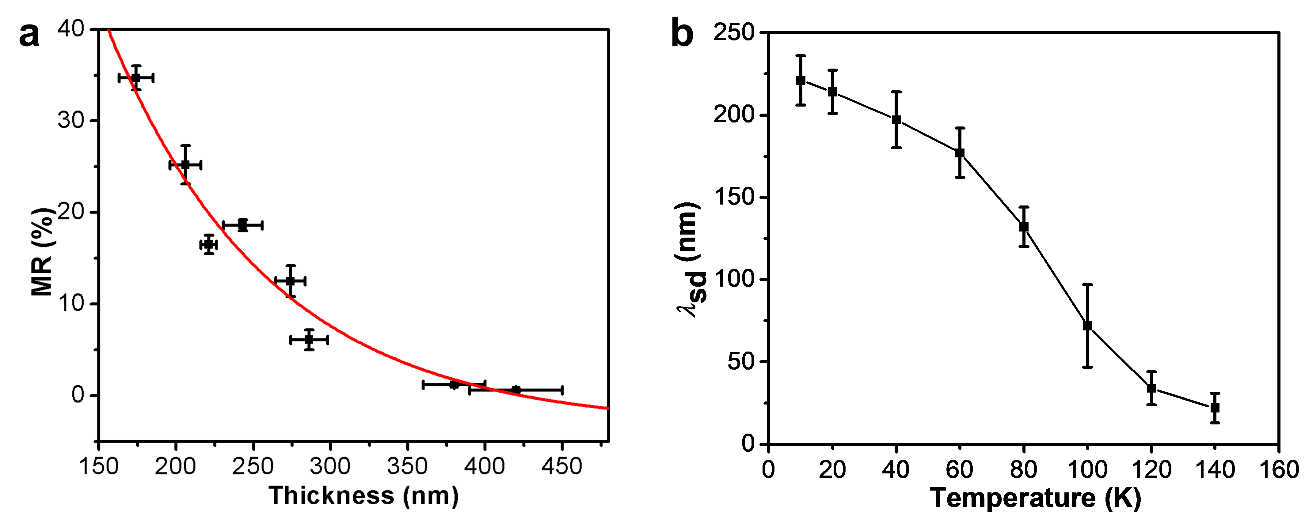
**

**Supplementary Figure 8 | Thickness dependece study of spin valves based on MAPbBr_3_.** **a,** GMR_max_ vs the MAPbBr_3_ interlayer thickness *d* obtained in various fabricated NP-MAPbBr_3_ based spin-valves measured at 10 K. Each point is given by a SV device with a certain thickness. The error bar of thickness is calculated from standard deviation of 3 thickness values measured near the device by profilometer. The error bar of MR signal is calculated from standard deviation of MR signal measured 5 times at the device. Red solid line is a fit using Supplementary Equation 3 which gives a spin diffusion length value of 220 nm. **b**, Temperature dependence of *λ*_sd_. The error bar of *λ*_sd_ is given by the fitting.

**Supplementary Note 1: Characterization of the MAPbBr_3_ thin film**

In order to improve the uniformity of our MAPbBr_3_ thin films, a technique called ‘nano-crystal pining’ has been applied during spin coating of the films. In this method chloroform solvent was drop-casted onto the perovskite film during spin coating.^1^ The nanocrystal pining process results in smaller MAPbBr_3_ grain size, thus reducing the pinholes in the film. Supplementary Figure 1 shows the SEM image and XRD study of MAPbBr_3_ with and without (as-grown) nanocrystal pining.

**Supplementary Note 2: Picosecond transient response of MAPbBr_3_ film**

Pump-probe transient absorption spectroscopy was performed using as pump a homemade Ti:Sapphire laser amplifier that operates at 1 kHz repetition rate, at 800 nm, and 150 fs pulse duration. The laser beam was split into two beams. One beam was subsequently frequency doubled to 3.1 eV with a pulse energy of 10 μJ using a BaBO_2_ nonlinear crystal and used as pump excitation, while the other was used to generate white light supercontinuum in an energy spectral range of 1.15 eV–2.7 eV that served as probe beam. The pump and probe beams were carefully adjusted to obtain complete spatial overlap. The transmission of the probe beam, *T*, was measured by a photodiode after dispersion by a monochromator in the absence of the pump, and at various time delays, *t* after the pump arrives at the sample. The photoinduced transmission change, *ΔT(λ,t)* was normalized by *T* to give the change in the optical density, *ΔT(λ,t)/T*. The samples were measured at temperature at 80K by transferring liquid nitrogen into the cryostat. Supplementary Figure 2 shows the spectrum and dynamics of the optical density change. The transient bleaching of the exciton last much longer than the maximum range of our translation stage, which indicates exciton lifetime in the range of tens of nanoseconds.

**Supplementary Note 3: Magnetic circular dichroism (MCD) of MAPbBr_3_ film**

The different absorption of left (σ+) and right (σ-) circular polarized light under high magnetic field is recorded in Faraday geometry. The high magnetic field up to 17.5 T was provided by a superconducting magnet (Maglab SCM cell 3). The wavelength of incident light from a tungsten lamp is tuned by a monochromator. The light is then circularly polarized by a combined linear polarizer and a photo elastic modulator (PEM) before its normal incidence on the perovskites film. The polarization of the light changed between left and right at PEM modulation frequency of 50 kHz. The transmitted light was collected by an optic fiber and sent into high frequency photodetector. The difference and sum of left and right circularly transmitted light is obtained by two lock-in with the same input from photodetector and different reference signals from PEM and a chopper. The MCD signal is then obtained as:

$MCD=\frac{T\left( \sigma+ \right)-T(\sigma-)}{T\left( \sigma+ \right)+T(\sigma-)}$ (1)

where *T(σ+)/ T(σ-)* is the intensity of left /right circular polarized transmitted light.

The magnetic field dependence of MCD signal is fitted by:^2^

$MCD=\frac{d\mathrm{Ln}(T)}{dE}\cdot\Delta E$ (2)

Where *T* is the transmission intensity, $\Delta E=g\mu_{B}B$ is the Zeeman splitting energy. As shown in Supplementary Figure 3b, we fitted the magnetic field dependence of MCD with Supplementary Equation 2, we can get the value of *g* factor=1.254.

**Supplementary Note 4: Additional contribution to the magneto-EL in the spin-LED device**

Despite the circular polarization of the EL emission due to the spin-polarized carriers in the spin-LED device, other effects, including Zeeman splitting and magnetic circular dichroism (MCD) of the window and LSMO layer can also contribute to the measured circular polarization shown in Figure 3c. In order to demonstrate that the obtained circular polarization in Figure 3c is mainly due to the injected spin carriers, we measured the field induced circular polarization (FICPO) caused by the Zeeman splitting of the exciton states, as well as possible field induced magnetic circular dichroism (MCD).^3^

For this we fabricated a traditional MAPbBr_3_-based LED device having similar structure as that of the spin-LED, except that we changed the FM electrode (LSMO) to a nonmagnetic ITO electrode, as shown in the inset to Supplementary Figure 4a. The EL circular polarization was measured by the same method with the same applied magnetic field value as that in the spin-LED at 10K. Supplementary Figure 4a demonstrates that the contribution of FICPO(*B*) and MCD(*B*) of the cryostat windows are below 0.1%, which is an order of magnitude smaller than the obtained EL circular polarization in the spin-LED.

Supplementary Figure 4b shows the MCD(*B*) response of the LSMO electrode and the window. This MCD(*B*) response was measured by monitoring the circular polarization of the PL in MAPbBr_3_ that travels through the LSMO electrode and cryostat window, at the same conditions as that of the spin-LED. The obtained MCD(*B*) response is about 0.1%, which is much smaller than what we measured in the spin-LED device. This experiment should not contradict the measured circular polarization of the PL at *B*=0 (3.1%), since here we measured the extra circular polarization caused by the field.

**Supplementary Note 5: Lack of inversion symmetry at MAPbBr_3_ surface**

Evidence of the inversion symmetry breaking may be obtained via Nonlinear Optics. We indeed observed second harmonic generation (SHG) emission at the MAPbBr_3_ surface, which is possible only when there is no inversion symmetry. This was achieved using pulsed fs laser system at 1.55 micron that is in the transparent region of the perovskite. When focusing on the MAPbBr_3_ surface we obtained (SHG) emission at 770 nm and third harmonic generation (THG) emission at 517 nm, as well as three-photon absorption induced PL at 550 nm, as seen in Supplementary Figure 5. In contrast, we only obtained THG emission when focusing inside the MAPbBr_3_ bulk crystal. Since SHG occurs only in materials that lack inversion symmetry, our measurements show that indeed the MAPbBr_3_ surface lacks inversion symmetry.

**Supplementary Note 6: Tunneling Magnetoresistance (TMR)**

As-grown MAPbBr_3_ thin films that do not undergo nanocrystal pinning exhibit totally different morphology. As shown in Supplementary Figure 6a, large crystal grains of several μm are formed in such MAPbBr_3_ films with pinholes that enable inter-diffusion of Co clusters; this may lead to LSMO/MAPbBr_3_/Co tunnel junction in the fabricated spin valve device.

Supplementary Figure 6b shows that a negative MR_max_ of 8% is obtained at 10 K in the SV device based on ‘as grown’ MAPbBr_3_ interlayer. The MR polarity changes compared to that of the GMR (Figure 4c) is striking, and points to a different MR origin here, which we identify as TMR. The TMR polarity change may originate from the interface geometry of the Co inter-diffusion clusters, which leads to different spin-polarized hybridization at the Co/MAPbBr_3_ interfaces. Similar TMR polarity change induced by pinholes was reported in LSMO-based magnetic tunnel junction.^4^

In contrast to the existence of the Hanle effect as measured in spin valve based on nanocrystal pinning MAPbBr_3_, no Hanle effect was observed in TMR-based device, since there is no spin-polarized current in the as grown MAPbBr_3_ interlayer. As shown in Supplementary Figure 6c, the resistance in the parallel and antiparallel configuration does not show an obvious change with out-of-plane magnetic field up to 60 mT.

**Supplementary Note 7: Possible artefacts in SV based on hybrid perovskites**

Possible artefacts in the measured GMR of the MAPbBr_3_-based SV devices that include tunneling anisotropic magnetoresistance (TAMR)^5^ and anisotropic magnetoresistance of the electrodes (AMR) are ruled out by the following control experiments.

We measured magnetoresistance in a device having LSMO/MAPbBr_3_/Au structure. No obvious TAMR response has been observed at 10K. The magnetoresistance of 0.5% is related to high field magnetoresistance (HFMR) or AMR of LSMO bottom electrode, as shown in Supplementary Figure 7a. This MR response does not show any similarity to that in the GMR(*B*) response measured in our SV of LSMO/MAPbBr_3_/Co. AMR(*B*) response was also measured for the LSMO bottom electrode. As shown in Supplementary Figure 7b, an AMR of 0.6% is measured at 10K, which is two order of magnitudes smaller than the GMR value measured in the SVs.

**Supplementary Note 8: Thickness dependence study of SV devices**

GMR(*B*) response of several NP-MAPbBr_3_ -based SV devices of different thicknesses, *d* were measured. The GMR_max_ value decreases steeply with the interlayer thickness, as summarized in Supplementary Figure 8a. We have used the modified Jullière model to estimate the spin diffusion length, $\lambda_{\mathrm{sd}}\mathrm{fr}$om the GMR_max_(*d*) dependence given by:^6^

GMR_max_*(d)* $\propto\frac{2P_{1}P_{2}exp(-(d-d_{0})/\lambda_{\mathrm{sd}})}{1-P_{1}P_{2}\exp(-(d-d_{0})/\lambda_{\mathrm{sd}})}$ (3)

where $P_{1}$ and $P_{2}$ are the spin polarization of the two FM electrodes, $d_{0}$ is the thickness of a possible “ill-defined” interlayer in the device, and $\lambda_{\mathrm{sd}}$ is the spin diffusion length. From the GMR(*d*) fit using Supplementary Equation 3 (red solid line in Supplementary Figure 8a), we obtained $P_{1}P_{2}=(19\pm4)\%$, $d_{0}=65\pm6 nm$, $\lambda_{\mathrm{sd}}=221\pm18 nm$ at 10K, which is a relatively long spin diffusion length. We repeated the GMR*(d)* measurements at different temperatures, and extracted the spin diffusion length vs temperature, assuming that $d_{0}$in Supplementary Equation 3 is temperature independent. The obtained $\lambda_{\mathrm{sd}}$*(T*) is plotted in the Supplementary Figure 8b.

**Supplementary References**

1. Cho, H. et al. Overcoming the electroluminescence efficiency limitations of perovskite light-emitting diodes. Science 350, 1222-1225 (2015).
2. Wu, Y. J., et al. Valley Zeeman splitting of monolayer MoS2 probed by low-field magnetic circular dichroism spectroscopy at room temperature. App. Phys. Lett. 112.15 (2018): 153105.
3. Fang, L. et al. Electrical spin injection from an organic-based ferrimagnet in a hybrid organic-inorganic heterostructure. Phys. Rev. Lett **106**, 156602 (2011).
4. Mukhopadhyay, S. & Das, I. Inversion of magnetoresistance in magnetic tunnel junctions: Effect of pinhole nanocontacts. Phys. Rev. Lett **96**, 026601 (2006).
5. Gould, C. *et al.* Tunneling anisotropic magnetoresistance: a spin-valve-like tunnel magnetoresistance using a single magnetic layer. Phys. Rev. Lett **93**, 117203 (2004).
6. Xie, S. J. et al. Ground-state properties of ferromagnetic metal/conjugated polymer interfaces. Phys. Rev. B **67**, 125202 (2003)
